# Protein Supplementation, Plasma Branched-Chain Amino Acids, and Insulin Resistance in Postmenopausal Women: An Ancillary Study from the Supplemental Protein to Outsmart Osteoporosis Now (SPOON) Trial

**DOI:** 10.3390/nu17132104

**Published:** 2025-06-25

**Authors:** Jessica Dauz Bihuniak, Alessandra Byer, Christine A. Simpson, Rebecca R. Sullivan, Josephine M. Dudzik, Karl L. Insogna, Jeannette M. Beasley

**Affiliations:** 1 Department of Nutrition and Food Studies, Montclair State University, Montclair, NJ 07043, USA; bihuniakj@montclair.edu; 2 Mather Medical Group, Northwell Health, Port Jefferson, NY 11777, USA; alessandra.byer16@gmail.com; 3 Department of Internal Medicine, Yale School of Medicine, New Haven, CT 06510, USA; christine.simpson@yale.edu (C.A.S.); becky.sullivan@yale.edu (R.R.S.); karl.insogna@yale.edu (K.L.I.); 4 Department of Nutrition and Food Studies, Steinhardt School of Culture, Education, and Human Development, New York University, New York, NY 10003, USA; dudzij01@nyu.edu; 5 Department of Medicine, NYU Grossman School of Medicine, New York, NY 10016, USA

**Keywords:** branched-chain amino acids, insulin resistance, postmenopausal women, dietary protein

## Abstract

**Background/Objectives**: Studies have reported an increased risk of type 2 diabetes among people with higher protein intake. Moreover, branched-chain amino acids (BCAA) are reported to be positively associated with insulin resistance (IR). However, it is not understood whether elevated levels of BCAA are causal to IR development, or if higher BCAA are a marker of IR. The objective of this study was to examine the effects of long-term protein and carbohydrate supplementation on plasma BCAA levels, and the relationship between plasma BCAA and IR in postmenopausal women. **Methods**: Stored samples and data from 84 postmenopausal women who participated in a protein supplementation trial (SPOON) were included. Exclusion criteria consisted of protein intakes less than 0.6 g/kg or greater than 1.0 g/kg, a body mass index (BMI) greater than 32 kg/m^2^ or less than 19 kg/m^2^ diseases, and conditions and medications known to impact musculoskeletal health. Subjects were randomized to a whey protein (PRO: *n* = 38) or maltodextrin supplement (CHO: *n* = 46) for 18 months. Plasma BCAA, homeostatic model assessment of insulin resistance (HOMA-IR) and body composition were analyzed at baseline and 18 months. **Results**: At baseline, there were no significant associations between plasma BCAA and IR. There were also no significant changes in plasma BCAA or IR by study arm. However, there was a significant positive association between plasma BCAA and IR in both groups at 18 months (CHO: r = 0.35, *p* = 0.02; PRO: r = 0.35, *p* = 0.03). **Conclusions**: Findings from this study warrant future research to examine other diet and lifestyle factors that may mediate the relationship between circulating BCAA and IR in postmenopausal women.

## 1. Introduction

The role of dietary protein in the development of type 2 diabetes (T2D) is an area of active investigation [1]. Higher protein intake is associated with increased satiety, higher thermogenesis and reduced caloric intake and has been studied for its role in the management of obesity and associated chronic diseases, including T2D [2,3]. However, findings from epidemiological studies have suggested a positive association between higher protein intake, meat consumption and incident T2D [2,4,5,6,7,8], despite protein’s role in enhancing satiety and diet-induced thermogenesis. The association of protein intake and risk of T2D has been studied in large populations from the United States, Australia, Europe and Japan, and included hundreds to thousands of T2D cases [7,9,10]. A pooled analysis of data from the Nurses’ Health Study I and II, and the Health Professionals Follow-up Study suggested animal protein was associated with higher T2D risk, whereas vegetable protein was associated with lower risk [9]. These results suggest that total protein and protein source may be related to the development of T2D. In fact, higher consumption of red meat has been associated with a higher risk of T2D [11]. Overall, it is unclear whether it is the type of protein, nutrient profile, cooking methods or a combination of characteristics of dietary protein that explain the association with T2D.

Data from three prospective cohort studies suggest total and animal protein intake are associated with higher risk of T2D [9]. One possible explanation for the relationship between dietary animal protein and T2D risk may be related to the higher branched-chain amino acid (BCAA) content of animal protein sources compared to vegetable protein sources. The BCAAs, leucine, isoleucine and valine, possess a unique structure in that they contain a branched aliphatic side chain, and are classified as essential, meaning they must be supplied by the diet [12]. Dietary sources include meat, chicken, fish, dairy, and eggs. Circulating levels of BCAAs have been positively associated with measures of insulin resistance (IR) [13,14,15]; however, it is unknown whether a dietary-protein-induced rise in circulating BCAAs are causal in the development of IR.

The potential link between BCAA and IR is of particular concern for populations at higher risk of developing T2D, one of which is postmenopausal women. While the prevalence of T2D is high among women of all ages, postmenopausal women have a higher risk for and incidence of T2D compared to those at premenopausal status [16,17]. Age of menopause onset, along with body mass index (BMI), waist circumference, lipid profile and presence of comorbid conditions, all contribute to T2D risk in this population [16,17,18]. As life expectancy in the US continues to increase and with the average age of menopause onset about 44.2 years [18], identifying modifiable risk factors for the development of T2D in this subgroup is critical for reducing the incidence of T2D and improving numerous long-term health outcomes.

The primary objective of this analysis was to examine the effects of a whey protein isolate, a supplement that contains BCAAs, on changes in plasma levels of BCAAs in postmenopausal women. The second objective was to evaluate the relationship between plasma BCAA levels and IR. We hypothesized that plasma BCAA levels would be associated with IR in participants receiving the protein supplement and that this association would not be observed in the control group. To our knowledge, this is the first study to utilize data from a tightly controlled, 18-month, randomized, controlled trial to examine the relationship between dietary protein, circulating BCAAs and insulin resistance in postmenopausal women.

## 2. Materials and Methods

### 2.1. Participants

The Supplemental Protein to Outsmart Osteoporosis Now (SPOON) study and study participants have been described fully elsewhere [19,20]. In brief, SPOON was an 18-month, randomized, double-blind, placebo-controlled trial among older adults that was conducted by researchers at Yale University School of Medicine, the University of Connecticut and the University of Connecticut Health Center. The objective of SPOON was to evaluate the impact of a whey protein supplement (Provon^®^ 290, Glanbia Nutritionals, Twin Falls, ID, USA) or an isocaloric maltodextrin control supplement (Maltrin^®^ M100, Grain Processing Corporation, Muscatine, IA, USA) on bone mineral density and body composition in postmenopausal women and older men. Women ≥ 60 years and men > 70 years with self-reported protein intake between 0.6 and 1.0 g/kg/d were included in SPOON. Men and women with self-reported protein intakes less than 0.6 g/kg or greater than 1.0 g/kg, or a body mass index (BMI) greater than 32 kg/m^2^ or less than 19 kg/m^2^ were excluded. Additional exclusion criteria consisted of diseases, conditions and medications known to impact musculoskeletal health. Postmenopausal women who completed the SPOON study (*n* = 84) and consented to having their biological samples stored for future research were selected for this ancillary study. BCAA were measured in a cohort of 84 female valid completers from SPOON. Subjects who consumed a minimum of 20 g of either the protein (*n* = 38, protein group, PRO) or maltodextrin (*n* = 46, carbohydrate group, CHO) supplement for 18 months were considered valid completers. The Institutional Review Boards of the University of Connecticut Health Center and Yale University School of Medicine approved this study.

### 2.2. Laboratory Measures

Fasting blood samples were collected by routine venipuncture at baseline and 18 months of SPOON, processed and stored at −70 °C. For this ancillary study, BCAA were measured from frozen plasma samples by the Yale Bone Center Mineral Metabolism Laboratory using a colorimetric assay kit (Abcam’s Branched-Chain Amino Acid Assay Kit, Cambridge, MA, USA). Fasting fingerstick glucose measurements were made during SPOON using the Novastrip glucose meter (Yale New Haven Hospital Research Unit, New Haven, CT, USA) or the Bayer Contour glucometer (University of Connecticut Health Center, Farmington, CT, USA) [19]. IR has been previously assessed in the subgroup of valid completers [20]. The HOMA2 calculator, published by the University of Oxford, was used to calculate insulin resistance from fasting insulin and serum glucose.

### 2.3. Data Analysis

Descriptive statistics summarized participant characteristics, plasma BCAA, and HOMA-IR by intervention assignment with mean (SD) for continuous scale variables and categorical variables presented with frequency distributions at baseline and 18 months. To assess normality of the variables, visual inspection of the histograms and Q-Q plots was used along with the Shapiro–Wilk’s test. Independent *t*-tests (for normally distributed data), Mann–Whitney tests (for non-normally distributed data) and chi-square tests (categorical data) were used for between group comparisons. Spearman correlations were used to evaluate the association between plasma BCAA and glucose, insulin, HOMA-IR, body composition variables and BMI. Bar plots and independent *t*-tests were used to evaluate comparability between the completers from SPOON (included in this ancillary study) and non-completers from SPOON (not included in this ancillary study) for demographic variables. Descriptive analyses, tests for normality, independent *t*-tests, Mann–Whitney test and Spearman correlations were conducted using IBM SPSS Statistics for Windows, version 24.0 (IBM Corp., Armonk, NY, USA). *p*-value < 0.05 was the criterion for statistical significance.

## 3. Results

### 3.1. Participant Characteristics

Demographic and health characteristics (age, education level, depression, cancer, heart disease, hypertension, smoking, alcohol consumption, weight and BMI) were similar between SPOON completers (included in this ancillary study) and non-completers (not included in this ancillary study; age and weight data presented in Supplemental Figures S1 and S2). Additionally, participant characteristics between CHO versus PRO supplementation groups were not statistically different (SPOON completers; Table 1). The sample was predominately white (94.0%) and not Hispanic (97.6%). Given the age of the study participants, average BMI for both groups was considered to be within a healthy range throughout the study period. Additional anthropometric and dietary data for this cohort of completers has been previously described [20].

### 3.2. Plasma BCAA and Insulin Resistance, Body Composition and BMI

Plasma BCAA and insulin resistance did not differ between the two supplementation groups at baseline or 18 months and remained relatively stable throughout the study (Table 2). At baseline, there were no significant associations between plasma BCAAs and HOMA-IR in either group (Table 3). However, there was a significant positive association between plasma BCAAs and HOMA-IR in both groups after 18 months (*p* = 0.02 in CHO group; *p* = 0.03 in PRO group). There was also a significant positive association between plasma BCAAs and fasting insulin in the CHO supplementation group after 18 months (*p* = 0.02). There was no association between plasma BCAA concentrations and β-cell function in either of the two groups. There were also no associations between BCAA concentrations and measures of lean mass or BMI.

## 4. Discussion

Among SPOON participants who consumed a minimum of 20 g of a whey protein isolate (PRO) or maltodextrin supplement (CHO) for 18 months, there were significant associations between plasma BCAAs and IR in both supplement groups at the end of the study. A significant association was also observed between plasma BCAAs and fasting insulin in the CHO group, but not in the PRO group. PRO group participants experienced biochemical changes that were in alignment with our hypothesis, while results from the CHO group were unexpected.

It is important to interpret observed relationships through a mechanistic lens by considering underlying biochemical pathways. The BCAAs, leucine, isoleucine, and valine, are essential amino acids primarily catabolized in skeletal muscle via the branched-chain α-keto acid dehydrogenase (BCKDH) complex. This enzymatic pathway is distinct from the hepatic metabolism that characterizes most other amino acids, and its function is tightly regulated by both nutritional and hormonal signals [21]. The primary hypothesized biological mechanism driving the association between higher protein intakes and cardiometabolic risk factors such as obesity and T2D is the continual activation of mTOR complex 1 (mTORC1) by circulating levels of BCAA [22]. The mTOR signaling network is responsible for several cellar processes, with mTORC1 regulating cell growth in response to a number of biological factors including amino acid availability. BCAA-mediated mTORC1 activation can lead to changes in insulin receptor functioning, which interferes with the normal action of insulin by disrupting insulin signaling leading to reduced glucose uptake and storage while increasing glucose production [23,24]. A limitation of this study is the absence of direct measurements of enzymatic activity or intermediary metabolites, which are necessary to determine whether elevated BCAA levels reflect increased intake, reduced catabolism, or altered flux through related metabolic pathways. Moreover, chronic nutrient overload may overwhelm normal enzymatic processing, particularly when micronutrient cofactors are suboptimal, leading to metabolic bottlenecks and altered energy homeostasis [5]. Therefore, the interpretation that elevated BCAA levels may contribute to insulin resistance should be framed within the broader concept of metabolic overload, rather than a direct causal pathway. Future studies would benefit from incorporating metabolomic, genomic and enzymatic profiling to disentangle these interactions and better define the role of BCAAs in metabolic health.

The present findings complement epidemiological studies that reported associations between BCAAs and impaired glycemic control in middle aged and older women. A prospective longitudinal analysis of data from the Women’s Health Initiative reported that higher BCAA intake was significantly associated with a higher risk of T2D [7]. Similarly, a cross-sectional analysis of the Women’s Health Study reported a significant, positive association between plasma BCAAs and insulin resistance in women aged 45 and over [25]. While these data support a role for dietary and circulating BCAAs in the development of impaired glucose tolerance, there is a lack of intervention research in older women to substantiate these findings. One randomized controlled trial (RCT) conducted in older women (aged > 65 y) found no effect of leucine supplementation on serum insulin, plasma glucose or IR [26]. However, this study differs from the present report in that the intervention duration was shorter (3 vs. 18 months), the sample size was smaller (*n* = 19 vs. *n* = 84), and the intervention supplemented only one BCAA, leucine, in combination with resistance training. Two short-term RCTs reported no effect of supplemental BCAAs on glycemic outcomes when delivered as either a whey protein supplement for 8 weeks [27] or as an essential amino acid supplement for 22 weeks [28]. Conversely, a short-term intervention study that included community-dwelling older men and women from England (average age 69 years) found beneficial effects of leucine-enriched whey supplementation on IR and serum insulin [29]. Similarly, a study in older Dutch adults (age ≥55 years) with obesity and T2D reported that the addition of a leucine-enriched whey protein supplement to a hypocaloric diet (600 kcal below estimated energy needs) plus exercise for 13 weeks resulted in improvements in fasting plasma insulin and IR [30]. To our knowledge, the current study is the first long-term RCT to assess the impact of whey protein supplementation on T2D risk in a large sample of postmenopausal women living in the US.

The current study specifically targeted postmenopausal women, an understudied population in the literature on BCAAs and IR. Postmenopausal women are also at higher risk for dysglycemia compared to men of the same age and premenopausal women [31,32]. The menopausal transition, along with chronological cellular aging, is a unique independent risk factor for IR as a result of decreased circulating estrogen and increased androgen levels during and after menopause [33]. This hormonal shift causes the increase of adipose tissue mass leading to the development of abdominal obesity, an independent risk factor for T2D [33,34]. As of 2022, approximately 19% of women aged 65 or older in the US have been diagnosed with diabetes, a higher prevalence compared to women or adults of any age (14.1% and 14.7%, respectively) [35]. Developing strategies to identify and mitigate risk factors for T2D in postmenopausal women is critical to reduce T2D prevalence in the coming years. Additional intervention research is needed to better understand the relationship between dietary factors, circulating BCAA and IR in postmenopausal women.

Despite no observed changes in body composition, weight and physical activity, there was an association between BCAAs and IR in our sample of postmenopausal women at 18 months that was not observed at baseline. This begs the question as to whether the relationship between circulating BCAAs and IR is due to aging. Given the tightly controlled study design, which controlled for plausible risk factors for IR, including diet, body composition, weight, physical activity and smoking status, we question whether the 18-month study period is a significant timeframe to capture age-related changes in the IR–BCAA relationship. However, there may be additional factors that influence this relationship that we did not account for such as changes in serum lipids [36] and protein turnover [12].

This study has some limitations. The sample was predominantly non-Hispanic White, which limits the generalizability of the study findings. Although our sample size is larger than that of prior studies summarized above, it is still considered small. The current study also has a number of strengths. To ensure dietary compliance and prevent weight gain from the addition of the supplement, supplement adherence and weight were carefully monitored by registered dietitians during the 18-month study period. Usual physical activity, which could have influenced the study results, remained consistent for the duration of the study [19].

## 5. Conclusions

In this sample of postmenopausal women residing in the US, circulating BCAAs were positively associated with IR. Future longitudinal studies are needed to identify dietary factors in postmenopausal women that can modulate this relationship.

## Figures and Tables

**Table 1:** Participant characteristics of postmenopausal completers by randomization assignment, Supplemental Protein to Outsmart Osteoporosis Now (SPOON).

	CHO *n* = 46	PRO *n* = 38	*p*-Value
Age, years, mean (SD)	69.3 (6.0)	68.9 (5.8)	0.8
BMI, v2, mean (SD)	25.8 (4.0)	26.0 (3.6)	0.8
BMI, v9, mean (SD)	26.0 (4.3)	26.0 (3.7)	0.9
Δ BMI, mean (SD)	0.19 (1.6)	0.01 (0.8)	0.3
Race, *n* (%)			0.4
White	44 (95.7)	35 (92.1)	
Black	1 (2.2)	2 (5.3)	
Asian	1 (2.2)	1 (2.6)	
Ethnicity, *n* (%)			0.5
Not Hispanic	44 (95.7)	38 (100)	
Hispanic	1 (2.2)	0 (0)	
Missing	1 (2.2)	0 (0)	

Abbreviations: BMI: body mass index (kg/m^2^); CHO: carbohydrate group; *n*: sample size; PRO: protein group; SD: standard deviation; CHO: carbohydrate supplementation arm; PRO = protein supplementation arm. Independent *t*-tests (for normally distributed data), Mann–Whitney tests (for non-normally distributed data) and chi-square tests (categorical data) were used for between group comparisons. *p*-value < 0.05 was the criterion for statistical significance.

**Table 2:** Plasma BCAA and insulin resistance of postmenopausal completers by randomization assignment, Supplemental Protein to Outsmart Osteoporosis Now (SPOON) ^a^.

	CHO (*n* = 46)		PRO (*n* = 38)	
	Baseline	18 Months	Baseline	18 Months
BCAA, umol/L	174 ± 40	174 ± 54	202 ± 73	196 ± 49
Glucose, mg/dL	90.2 ± 11.9	94.1 ± 14.7	91.8 ± 12.2	92.7 ± 10.9
Insulin, µU/mL	12.2 ± 5.0	11.8 ± 4.8	13.3 ± 5.9	13.4 ± 4.4
HOMA-IR, %IR^2^	1.6 ± 0.6	1.5 ± 0.6	1.7 ± 0.7	1.7 ± 0.6
HOMA-IR, %β^2^	132.8 ± 47.9	117.1 ± 41.8	137.0 ± 53.3	133.3 ± 37.5

^a^ All data presented as mean ± SD. Abbreviations: BCAA: branched-chain amino acids; CHO: carbohydrate group; HOMA-IR: homeostatic model assessment for insulin resistance; *n*: sample size; PRO: protein group. The HOMA2 calculator, published by the University of Oxford, was used to calculate insulin resistance from fasting insulin and serum glucose.

**Table 3:** Spearman correlations between Plasma BCAA and insulin resistance, body composition and BMI ^a^.

	CHO (*n* = 46)		PRO (*n* = 38)	
	Baseline	18 Months	Baseline	18 Months
Glucose, mg/dL	0.28 (0.06)	0.23 (0.13)	0.29 (0.08)	0.22 (0.18)
Insulin, µU/mL	0.26 (0.08)	0.35 (0.02) ^b^	0.25 (0.13)	0.32 (0.05)
HOMA-IR, %IR^2^	0.27 (0.07)	0.35 (0.02) ^b^	0.27 (0.1)	0.35 (0.03) ^b^
HOMA-IR, %β^2^	0.02 (0.88)	0.15 (0.33)	0.05 (0.79)	0.03 (0.85)
Lean Mass	0.07 (0.63)	−0.22 (0.14)	−0.14 (0.41)	−0.02 (0.89)
Trunk Lean Mass	−0.04 (0.79)	−0.26 (0.08)	−0.06 (0.71)	0.17 (0.32)
BMI	0.22 (0.15)	0.23 (0.13)	−0.06 (0.73)	−0.06 (0.73)

^a^ Data presented as correlation coefficient, r (*p*-value).^b^ *p* ≤ 0.05 is statistically significant. Abbreviations: BMI: body mass index (kg/m^2^); CHO: carbohydrate group; HOMA-IR: homeostatic model assessment for insulin resistance; *n*: sample size; PRO: protein group. The HOMA2 calculator, published by the University of Oxford, was used to calculate insulin resistance from fasting insulin and serum glucose.

## Data Availability

Data are available upon reasonable request to the corresponding author.

## Supplementary Materials

The following supporting information can be downloaded at: https://www.mdpi.com/article/10.3390/nu17132104/s1, Figure S1: Comparison of age (years) between Supplemental Protein to Outsmart Osteoporosis Now (SPOON) study valid completers and non-completers; Figure S2: Comparison of weight (kg) between Supplemental Protein to Outsmart Osteoporosis Now (SPOON) study valid completers and non-completers.
