# Supplementary material for: Protein Supplementation, Plasma Branched-Chain Amino Acids, and Insulin Resistance in Postmenopausal Women: An Ancillary Study from the Supplemental Protein to Outsmart Osteoporosis Now (SPOON) Trial
Source: Nutrients. 2025 Jun 25;17(13):2104. doi: 10.3390/nu17132104 (PMC12250933; doi:10.3390/nu17132104)
Supplement: Supplementary file 1 [file nutrients-17-02104-s001.zip › nutrients-3669932-supplementary.pdf]

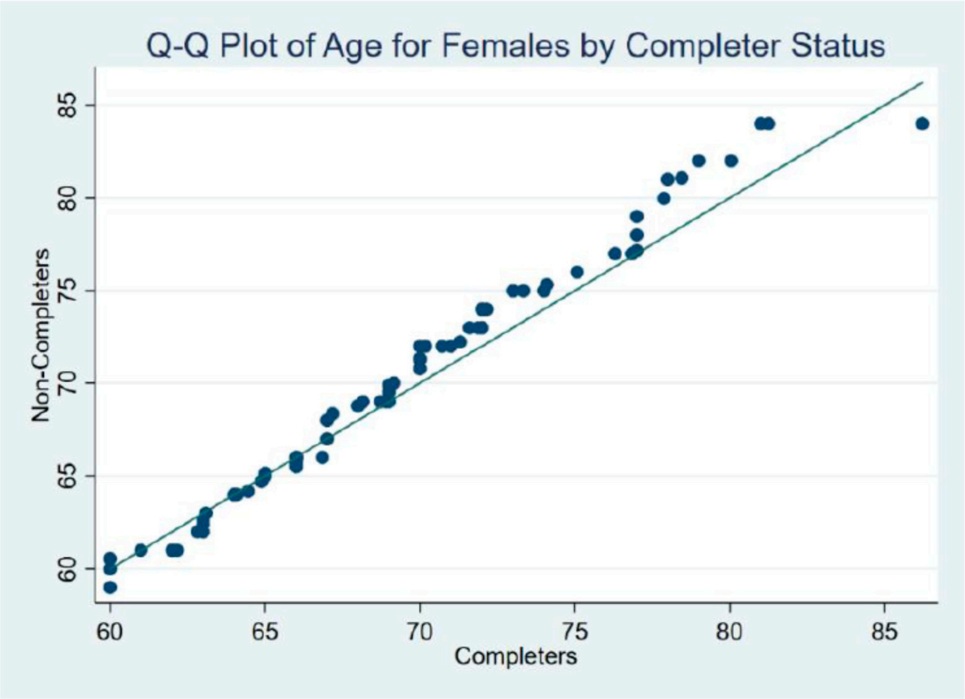

Supplemental Figure S1. Comparison of age (years) between Supplemental Protein to Outsmart Osteoporosis Now (SPOON) study valid completers and non-completers.

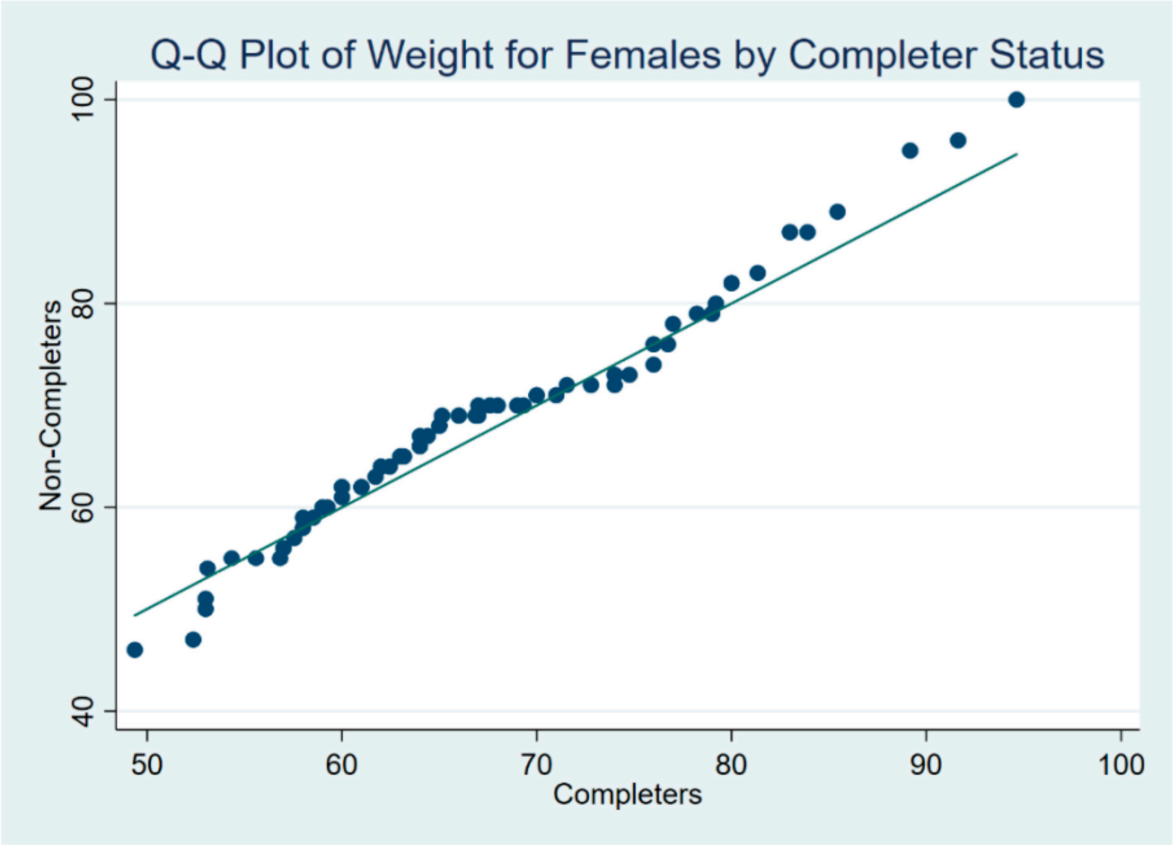

Supplemental Figure S2. Comparison of weight (kg) between Supplemental Protein to Outsmart Osteoporosis Now (SPOON) study valid completers and non-completers.
